# Comparability of a provisioned device versus bring your own device for completion of patient-reported outcome measures by participants with chronic obstructive pulmonary disease: qualitative interview findings

**DOI:** 10.1186/s41687-022-00492-5

**Published:** 2022-08-04

**Authors:** Louise Newton, Oliver Knight-West, Sonya Eremenco, Stacie Hudgens, Mabel Crescioni, Tara Symonds, David S. Reasner, Bill Byrom, Paul O’Donohoe, Susan Vallow, the Patient-Reported Outcome (PRO) Consortium, Electronic Clinical Outcome Assessment (eCOA) Consortium

**Affiliations:** 1 Clinical Outcomes Solutions, Folkestone, UK; 2 Broughton Life Sciences, Earby, UK; 3 Critical Path Institute, Tucson, USA; 4 Clinical Outcomes Solutions, Tucson, USA; 5 College of Public Health, University of Arizona, Tucson, AZ USA; 6 Imbria Pharmaceuticals, Boston, MA USA; 7 Signant Health, London, UK; 8 Medidata Solutions, London, UK; 9 Novartis Pharmaceuticals, East Hanover, NJ USA

**Keywords:** BYOD, ePRO, PRO measure, Bring your own device, Patient-reported outcome, Provisioned device, Qualitative, Equivalence, Comparability

## Abstract

**Background:**

There is interest in participants using their own smartphones or tablets (“bring your own device”; BYOD) to complete patient-reported outcome (PRO) measures in clinical studies. Our study aimed to qualitatively evaluate participants’ experience using a provisioned device (PD) versus their own smartphone (BYOD) for this purpose.

**Methods:**

Participants with chronic obstructive pulmonary disease (COPD) were recruited for this observational, cross-over study and completed PRO measures daily on one device type for 15 days, then switched to the other device type to complete the same measures for another 15 days. After each 15-day period, semi-structured interviews were conducted about their experience with the device.

**Results:**

Of 64 participants enrolled, the final qualitative analysis populations comprised those who participated in an interview without protocol violations. Thus, the qualitative longitudinal population (LP) included n = 57 (89%), while the qualitative cross-sectional population (CSP) included n = 60 (94%). CSP participants found both device types easy to use. Twenty CSP participants (33%) reported missing data entry on at least one day when using PD, and 24 (40%) reported missing at least one day when using BYOD. In the LP, preference for one of the device types was somewhat evenly split; 45.6% (n = 26) preferred PD and 50.9% (n = 29) preferred BYOD. The most common reason for preferring PD was that it was “dedicated” to the study; the “convenience” of carrying a single device was the main reason for preferring BYOD.

**Conclusion:**

The findings from the interviews demonstrated few differences in participants’ experience completing PRO measures on a PD versus BYOD. Our study supports the use of BYOD as a potential addition to PD for collecting PRO data and contributes evidence that BYOD may be employed to collect PRO data in demographically diverse patient populations.

## Background

Collecting patient-reported outcome (PRO) data is often an integral part of clinical research. Historically, PRO measures had been administered on paper, but the past 20 years has seen an increase in collecting these data electronically; this shift has led to a substantial body of evidence supporting the measurement equivalence of paper and electronic PRO (ePRO) modes [1]. ePRO data collection can help minimize missing data and data entry errors, increase response compliance, as well as allow for time-stamping of records and assurance that PRO entries are made within appropriate recall periods, as specified by the protocol [2]. The latter is particularly important for verifying protocol adherence in the use of patient diaries completed outside of clinical/researcher supervision.

Over the last decade, PRO measures have been programmed in software applications (“apps”) on electronic devices which are provided to study participants (provisioned devices [PD]) and then returned to the study site at the end of the trial. There has also been a corresponding increase in consumer smartphone ownership capable of running similar apps. Increased personal smartphone ownership has generated considerable interest in having participants use their own devices (‘bring your own device’ [BYOD]) in clinical trials and other studies. There is a belief that a BYOD approach may help reduce drug development costs (primarily because fewer PDs need to be supplied) and may be more convenient for study participants. Importantly, high data security is available for data storage and transfer for both PD and BYOD. Additionally, studies on mode equivalence from paper to a variety of electronic formats support the hypothesis that small format changes between devices may not impact the data collected [3].

In previous studies assessing the feasibility of using BYOD for ePRO data collection, high compliance was found, and most participants did not find the process of completing daily items burdensome [3–6]. In another study, participants (n = 155) with chronic health conditions that caused daily pain or discomfort responded very similarly to PRO measures completed on paper, their own smartphone, and a PD during a single site visit. Nearly all participants reported that they would definitely or probably be willing to download an app to their own smartphone for a clinical trial [3]. A study that randomized participants (n = 87) to receive a 28-day intervention for medication adherence on either their own smartphone or a PD reported that PD users showed higher initial adherence rates, but greater decline than those participants using their own smartphone over the study period. Qualitative interviews with a subset of the study sample (n = 10) indicated that participants in both the BYOD and PD groups preferred to use their own smartphone in future research [7].

Given these research findings, there is a need to explore the potential for BYOD to collect PRO data in clinical research, in a repeated measures cross-over design, where participants use both their own smartphone and a PD at home over multiple days. Such a study allows for not only the quantitative assessment of score equivalence and compliance between device types, but also a qualitative investigation of participants’ experience and potential device preference. This paper describes the qualitative interview findings. The quantitative equivalence and compliance findings will be published separately.

## Methods

### Study design and participants

This observational, mixed methods, cross-over study was designed to explore participants’ real-world experiences of using BYOD and PD to collect PRO data both daily and weekly, as well as to understand preference (if any) for device type. Participants were recruited from four clinical sites in the United States (Chicago, St. Louis, New Orleans, Baltimore). Purposive sampling was employed to recruit sufficient numbers of suitable individuals into this study. Participants were required to own an Apple (iOS) or Android smartphone less than three years old which passed a web-based specification test to confirm that the operating system, hardware, and screen size of the smartphone were sufficient to run the app. Participants were required to be at least 40 years old, have a clinical diagnosis of COPD, and unlikely to experience any change in clinical status or treatment during the study period. In order to evaluate if BYOD has potential with populations that are less technology-oriented, we chose to recruit adults with COPD in part because of their relatively older age and, therefore, potential to be less familiar with smartphones.

The study was approved by US Copernicus Group Independent Review Board®. All participants provided written informed consent prior to taking part in the study.

### Study procedures and PRO measures

The app used in this study was programmed by ERT® (Pittsburgh, PA, USA, now named Clario) who also supplied the study PDs. The PD was an Android smartphone (Samsung Galaxy 4 running Android v4.4.4) with a 5″ touchscreen. Three PRO measures (EXAcerbations of Chronic pulmonary disease Tool [EXACT®], COPD Assessment Test™ [CAT], patient global impression of severity [PGIS]) were programmed into the app, which was available for participants to install on their own devices through either the Google Play Store (Android-based phones) or the Apple App Store (Apple iOS-based phones). The PDs came preinstalled with the Android version of the app.

The EXACT® is a daily diary used to quantify and measure exacerbations of COPD developed specifically to be administered electronically [8]. The EXACT® was available for completion on all study days and intended to be completed each night. The CAT was developed as a single-page, paper-based PRO measure of the impact of COPD on a person’s life. In this study, the CAT was reformatted such that each item was presented on a separate screen. The PGIS was also programmed into the app for participants to report the perceived severity of their COPD symptoms over the previous seven days. The CAT and PGIS were available for completion on Day 1, 8, 15, 16, 23, and 30. Instructions and formatting of the PRO measures were the same across device types; any visual differences were due to the size of the screen of the BYOD device and in native operating system graphical styles (e.g., in the placement of hard coded fonts such as frame headers).

There were three study visits in total. At the first visit, participants were allocated to either Group A or B. Group A received a PD with the app pre-installed and Group B was assisted by study staff with downloading the app to their own smartphone. Both groups were trained by site staff on how to access and use the app to complete the PRO measures. Site staff helped participants set up a time for completing the PRO measures each evening at the participant’s preferred bedtime; participants could complete the diary within a six-hour window, which was scheduled to be three hours before and after their selected bedtime. A reminder to complete the diary was programmed to sound each day one hour before participants’ bedtime, at their bedtime, and one hour later, unless all PRO measures were completed before any reminder timepoints. It was possible for participants to disable reminders for BYOD through their device settings, although participants were not informed how to do this. Reminders could not be disabled for the PD. Training also included a practice feature within the app itself and the dissemination of a quick reference guide (QRG) for each device type which covered how and when to complete the PRO measures. The ePRO vendor also staffed a Help Desk which participants were able to call any time if they had queries or difficulties during the study.

At the second visit, which was up to five days after Day 15, participants took part in a 60-min face-to-face semi-structured qualitative interview. Interviews were conducted by a trained qualitative interviewer using a semi-structured discussion guide. Participants were asked to discuss the ease of use/overall experience, their capacity to complete PRO measures as intended, reasons for not completing them as intended, problems with the device not functioning correctly, usefulness of training and QRG, perceptions of data security, and interruptions while completing the measures (e.g., due to telephone calls, messages, notifications from other apps). Participants were also asked to assess ease of use/overall experience quantitatively using an 11-point numeric rating scale where 0 = very difficult/poor and 10 = very easy/good. After the interview, participants were assigned to the other device type and trained to use it. The next period of the study involved the participant completing the same PRO assessment schedule for 15 days on the other device type.

At the final visit, participants completed a second interview about their experiences of completing the PRO measures using the alternate device type (topics covered were the same as the first interview) as well as their preference, if any, for PD or BYOD.

### Data analysis

Transcribed data from the qualitative interviews were entered into ATLAS.ti (version 7.1). The interview data were analyzed by grouping discrete thematic codes into concepts and then further collating them into domains. Device preference was explored both qualitatively and quantitatively. A separate paper describes the results of the quantitative comparisons of equivalence and compliance across the device types.

## Results

### Study population

A total of 123 candidates diagnosed with COPD were screened for eligibility for the study. Of these, 59 were unable to be enrolled into the study. The most common reason for non-participation was a lack of FEV_1_ data available in the potential participant’s medical record (n = 25, 42.4%). BYOD-related reasons (either not owning a smartphone or not wanting to use it in this study) accounted for 14 (23.7%) of the 59 who could not take part in the study.

Sixty-four participants were enrolled into this study. Enrolled population (EP) participants had a mean age [SD] of 59.0 [10.55] ranging from 49 to 85 years. EP participants were mainly Black/African American (n = 33, 51.6%) or White (n = 26, 40.6%), and over half were female (n = 42, 65.6%), with a range of educational ability and work status (Table 1). Demographic characteristics were similar among the three participants that did not provide qualitative data and the rest of the EP.

**Table 1:** Demographic and clinical characteristics

Demographic or clinical item	Enrolled population (EP)		
	Group A (PD 1st)	Group B (BYOD 1st)	Total
	N = 23	N = 41	N = 64
Mean age (SD)	57.5 (11.33)	59.8 (10.13)	59.0 (10.55)
Min–Max	40–75	40–77	40–77
Gender			
Female	14 (60.9%)	28 (68.3%)	42 (65.6%)
Male	9 (39.1%)	13 (31.7%)	22 (34.4%)
Race			
White	13 (56.5%)	13 (31.7%)	26 (40.6%)
Black/African American	6 (26.1%)	27 (65.9%)	33 (51.6%)
Other	4 (17.4%)	1 (2.4%)	5 (7.8%)
Ethnicity			
Hispanic/Latino	4 (17.4%)	1 (2.4%)	5 (7.8%)
Not Hispanic/Latino	18 (78.3%)	40 (97.6%)	58 (90.6%)
Do not wish to state	1 (4.8%)	0	1 (1.7%)
Education			
Did Not complete high school	0	5 (12.2%)	5 (7.8%)
High school diploma	8 (34.8%)	12 (29.3%)	20 (31.3%)
Some college or certificate program	6 (26.1%)	13 (31.7%)	19 (29.7%)
College or university degree	9 (39.1%)	8 (19.5%)	17 (26.6%)
Graduate degree	0	3 (7.3%)	3 (4.7%)
Work status			
Employed full-time	15 (65.2%)	11 (26.8%)	26 (40.6%)
Employed part-time	2 (8.7%)	4 (9.8%)	6 (9.4%)
Homemaker	1 (4.3%)	1 (2.4%)	2 (3.1%)
Retired	2 (8.7%)	12 (29.3%)	14 (21.9%)
Unemployed	0	3 (7.3%)	3 (4.7%)
Disabled/on disability	3 (13.0%)	9 (22.0%)	12 (18.8%)
Other	0	1 (2.4%)	1 (1.6%)
Gold Stage*			
II	16 (69.6%)	30 (73.2%)	46 (71.9%)
III	6 (26.1%)	10 (24.4%)	16 (25.0%)
IV	1 (4.3%)	1 (2.4%)	2 (3.1%)
Participant-reported overall health			
Very good	3 (13.0%)	8 (19.5%)	11 (17.2%)
Good	16 (69.6%)	17 (41.5%)	33 (51.6%)
Fair	3 (13.0%)	15 (36.6%)	18 (28.1%)
Poor	1 (4.3%)	1 (2.4%)	2 (3.1%)
Clinician-reported COPD severity			
Very mild	0	1 (2.4%)	1 (1.6%)
Mild	0	13 (31.7%)	13 (20.3%)
Moderate	18 (78.3%)	18 (43.9%)	36 (56.3%)
Severe	5 (21.7%)	8 (19.5%)	13 (20.3%)
Very severe	0	1 (2.4%)	1 (1.6%)

*BYOD* Bring your own device, *PD* provisioned device, *COPD* chronic obstructive pulmonary disease

*GOLD stage II = FEV1 value is between 50 and 79% normal, GOLD stage III = FEV1 value is between 30 and 49% normal, GOLD stage IV = FEV1 value is < 3% normal

While the intention was to allocate an equal number of participants to each group, nine (14.1%) of the PDs encountered technical issues during enrollment, resulting in 23 participants (35.9%) being allocated to Group A (enrolled on PD initially) and 41 participants (64.1%) being allocated to Group B (enrolled on BYOD initially). The two allocation groups (Group A or B) were of similar mean [SD] ages (Group A = 57.5 years [11.33]; Group B = 59.8 years [10.13]) and gender split was similar to the EP (Group A n = 14/23 female, 60.9%; Group B n = 28/41 female, 68.3%). Race distributions among the two allocation groups did not reflect the EP. More of Group A participants were White (n = 13/23, 56.5%) whereas more of Group B participants were Black/African American (n = 27/41, 65.9%). Other demographic characteristics are summarized in Table 1.

Of the total EP (n = 64), most participants reported that they were “very much comfortable” using technology (n = 30, 46.9%) and specifically using a mobile phone (n = 40, 62.5%; Table 2). A higher proportion of participants in Group A reported they were “very much comfortable” with both technology and mobile phones.

**Table 2:** Participant experience with technology

Technology item	Enrolled population (EP)		
	Group A (PD 1st)	Group B (BYOD	Total
	N = 23	1st) N = 41	N = 64
Comfort using own smart phone			
Not at all comfortable	0	1 (2.4%)	1 (1.6%)
A little bit comfortable	1 (4.3%)	3 (7.3%)	4 (6.3%)
Somewhat comfortable	0	8 (19.5%)	8 (12.5%)
Quite A bit comfortable	5 (21.7%)	6 (14.6%)	11 (17.2%)
Very much comfortable	17 (73.9%)	23 (56.1%)	40 (62.5%)
Comfort using technology			
Not at all comfortable	1 (4.3%)	5 (12.2%)	6 (9.4%)
A little bit comfortable	2 (8.7%)	3 (7.3%)	5 (7.8%)
Somewhat comfortable	2 (8.7%)	11 (26.8%)	13 (20.3%)
Quite A bit comfortable	3 (13.0%)	7 (17.1%)	10 (15.6%)
Very much comfortable	15 (65.2%)	15 (36.6%)	30 (46.9%)
Smartphone type			
Apple iOS	10 (43.5%)	13 (31.7%)	23 (35.9%)
Android OS	13 (56.5%)	28 (68.3%)	41 (64.1%)

*BYOD* Bring your own device, *PD* Provisioned device

Two-thirds of the EP (n = 41, 64.1%; Group A n = 13, 31.7%; Group B n = 28, 68.3%) used an Android smartphone as their BYOD device; the remaining n = 23 (35.9%) used an Apple iPhone (Table 3). The specification of BYOD devices varied with the smallest and largest devices amongst the Android BYOD devices, where screen size and resolution varied from 4-inch screen (480 × 800 pixels screen resolution) to 5.7-inch screen (1440 × 2560 pixels screen resolution), among those devices for which the make and model were easily identifiable.

**Table 3:** Participant BYOD smartphone specification summary

Smartphone	Screen resolution	Screen size	Total enrolled population (EP) N = 64
iOS (n = 23)			
iPhone 5/5c	640 × 1136	4-inch	2 (3.1%)
iPhone 6/6 s	750 × 1334	4.7-inch	11 (17.2%)
iPhone 7	750 × 1334	4.7-inch	5 (7.8%)
iPhone 7 plus	1080 × 1920	5.5-inch	1 (1.6%)
Model not determined	–	–	4 (6.2%)
Android (n = 41)			
LG G4	1440 × 2560	5.5-inch	1 (1.6%)
LG K7	480 × 854	5-inch	2 (3.1%)
Motorola Moto G4	1080 × 1920	5.5-inch	2 (3.1%)
Samsung Note 5	1440 × 2560	5.7-inch	1 (1.6%)
Samsung Prevail 2	480 × 800	4-inch	1 (1.6%)
Make/model not determined	–	–	34 (53.1%)

As noted above, nine EP participants (14.1%) were due to be allocated to Group A initially (using PD) but experienced technical issues during the enrollment visit and were, therefore, allocated to Group B (enrolled on BYOD). All nine were from the same study site and all of the PDs supplied to the site did not function correctly; either the PD did not hold the battery charge (n = 5, 7.8%), the PD displayed the error message “EXE not recognized” indicating a problem with the PD recognizing the App (n = 2, 3.1%), or there was a problem connecting the PD to the local wi-fi (n = 2, 3.1%). The study site received new PDs to allow these participants to switch allocation group after the initial 15 days. No technical issues were reported in relation to the new PDs.

Finally, while the total EP consisted of 64 participants, due to drop-out, protocol violations, or some participants attending only one interview, the qualitative cross-sectional analysis population (CSP) included a total of 60 EP participants (94%) (i.e., completed at least one interview without protocol violations). Furthermore, 57 EP participants (89%) completed both interviews without protocol violations to form the qualitative longitudinal analysis population (LP).

### Interview results

#### What aspects of the PD/BYOD did participants like and/or dislike?

Ease of use and overall experience were rated equally positively across device types (Group A mean score: PD = 9.4, BYOD = 9.5; Group B mean score: PD = 9.7, BYOD = 9.5).

In the CSP (n = 60), many reported that the app itself was “easy,” “simple,” or “straightforward” to complete on the PD (n = 23, 38.3%) and BYOD (n = 20, 33.3%):

*“*It's just so simple and I don't have to write nothing down. It's right in the device. Super easy. … No problems whatsoever.” – Participant A, a Group A participant with an Android smartphone talking about PD

In relation to the devices themselves, 18 CSP participants (30.0%) liked that the PD had a “sole” or “dedicated” purpose because it meant they could not be interrupted by messages or notifications (n = 10, 16.7%) and the PD was not “cluttered” with other apps on the screen (n = 7, 11.7%). In contrast, many CSP participants liked the convenience of completing the app on BYOD because they always have their own smartphone with them (n = 13, 21.7%), thereby negating the need to carry and monitor an additional device (n = 17, 28.3%):

“I would rather have my phone.…I mean that way I don't have to keep up with somebody's else's stuff and my phone stays right beside me all the time. So, you know-…I don't have to keep up with several different devices.” – Participant B, a Group A participant with an iOS smartphone talking about BYOD

Table 4 summarizes additional features that participants liked about completing PRO measures on the PD and BYOD.

**Table 4:** Aspects of provisioned device and bring your own device that CSP participants liked

Which overall aspects did participants like about…?	Total CSP N = 60	
	…the PD	…the BYOD
Easy to use	23	20
Convenience of being own phone	N/A	25
Simple to navigate/find on device	10	13
Dedicated device (“sole purpose”)	18	N/A
Made participant more aware of COPD	5	12
App content was relevant	3	11
Good way to capture data	6	4
Device was quick/responsive	7	0
Font and buttons were good size	2	0
Made them want to quit smoking	0	2

Five CSP participants who started on BYOD (8.3%) (n = 4, 6.7% using Android and n = 1, 1.7% using iOS) described difficulty downloading the app on to their smartphone, in part due to problems accessing the local wi-fi or because the app store requested credit card information.

One CSP participant (1.7%) described her experience of using her iOS smartphone to access the app as slower than when using the PD because she had too many apps already installed on her own device. Two CSP participants (3.3%) with older Android smartphones discussed that they felt their own touchscreen was not as responsive as the PD.

Aspects about the PD disliked by CSP participants included that the battery drained too quickly (n = 7, 11.7%) and the requirement to use another device (n = 5, 8.3%).

“I had to keep it charged all the time and ...be aware of it. So it was like a little job every night... I found it a little cumbersome.” – Participant C, a Group A participant with an iOS smartphone talking about PD

A small number of CSP participants (n = 3, 5.0%) felt that the PD was “slow” in terms of its performance and loading different screens, that the screen was too small (n = 2, 3.3%), or the PD was too heavy and cumbersome.

“With the iPhone I'm used to quicker. It seems like everything comes on. This had a little more, took longer to cycle through things.” – Participant C, a Group A participant with an iOS smartphone talking about PD

Finally, four participants (6.7%) (PD n = 1, 1.7%; BYOD n = 3, 5.0%) commented they did not like that when they contacted the Help Desk, assistance was not given, or they were asked to call back the next day.

#### Did participants report that they missed a completion window for the daily diary at any time during the study and, if so, for what reasons?

Twenty CSP participants (33.3%) said they had missed at least one day when using the PD and 24 (40.0%) reported missing at least one day when using their own smartphone. Most CSP participants reported missing just one day (PD n = 16, 26.7%; BYOD n = 18, 30.0%), with eight CSP participants (13.3%) noting that they had missed two days (PD n = 3, 5.0%; BYOD n = 5, 8.3%), one (1.7%) reporting missing three days when using BYOD, and one (1.7%) reporting missing “four or five” days when using the PD. Of the 57 LP participants, eight participants (14.0%) commented that they missed at least one completion window when using both devices.

There were no unique device-related reasons given by CSP participants for not completing the diary during each completion window as instructed. The most frequently reported reasons were due to planned or unplanned social engagements (PD n = 7, 11.7%; BYOD n = 6, 10.0%); poor health or feeling unwell including hospitalization (PD n = 6, 10.0%; BYOD n = 5, 8.3%) that resulted in CSP participants either being hospitalized without their device or forgetting to complete it; or tiredness/falling asleep (PD n = 4, 6.7%; BYOD n = 7, 11.7%):

“I went to sleep and, um, didn't get a chance to do the questionnaire, and when I did wake up, it was after the time frame. And I tried, don't think I didn't.” – Participant D, a Group A participant with an Android smartphone talking about missing days on BYOD

A further 10 CSP participants (16.7%) (PD n = 5, 8.3%; BYOD n = 5, 8.3%) noted that they simply forgot about completing the diary. Some CSP participants missed the diary’s completion window because of difficulty either accessing the app or technical issues completing the diary (PD n = 2, 3.3%; BYOD n = 5, 8.3%) which they were unable to resolve on their own or with Help Desk support before the completion window closed.

Finally, low battery or power was reported by four CSP participants (6.7%) (PD n = 2, 3.3%; BYOD n = 2, 3.3%) as the reason they missed completing the diary; two CSP participants (3.3%) noted that the short battery life of the PD meant they were unable to complete the diary on one occasion. Another two CSP participants (3.3%) reported that they missed a day because they had not charged their BYOD.

#### How did participants interact with the reminder notifications?

Over half of CSP participants reported receiving the reminder notifications and finding them a useful aid to completing the PRO measures each evening, both on the PD (n = 36, 60.0%) and BYOD (n = 37, 61.7%).

However, two CSP participants (3.3%) commented that when using their own smartphone to complete the PRO measures, the notifications were of little value because the only time they looked at their smartphone was if it was ringing. In addition, several CSP participants explained they did not receive a notification when using PD (n = 18, 30.0%) or their own smartphone (n = 9, 15.0%), and a further 12 CSP participants (20.0%) were unsure but thought they may have completed the diary before the reminder was scheduled to be received. None of the CSP participants reported disabling these reminders when completing the PRO measures on their own smartphone.

Almost half of the CSP reported that they set their own reminder, in addition to the programmed notification (PD n = 15, 25.0%; BYOD n = 14, 23.3%), so that the diary’s completion window was not easily missed or simply as an extra precaution against forgetting. Typically, this process involved setting the alarm function on their own smartphone, setting an alarm clock, having physical reminders (such as written notes), or asking a family member to remind them, or a combination of the above:

“I put an alarm in my phone and make sure that I would do it every single time at the same time. Interviewer: Oh, so an alarm in your other phone? Participant: My-yes. Uh, it had like uh, it had like an um, alert to let me know but my phone had an alert, too. ..Because sometimes I tend to forget things and I'm like, I know I have to do this.” – Participant E, a Group B participant with an Android smartphone talking about setting own reminder.

To improve the functionality of the reminder notifications, some CSP participants (n = 11, 18.3%) suggested that they be louder, longer, or more “visual” as well as audible on both device types.

#### Did participants experience any interruptions when using their own smartphone to complete the PRO measures and what impact did this have on participants’ experience of completing the PRO measures?

Seventeen CSP participants (28.3%) reported that while completing the diary on their own smartphone they received a phone call (n = 11, 18.3%), a text message (n = 2, 3.3%), a phone call and a text message (n = 3, 5.0%), or a notification from another application (n = 1, 1.7%). Most CSP participants (n = 11, 18.3%) always ignored the interruption and completed the diary, and most (n = 9, 15.0%) received only one or two interruptions during the 15-day period. Importantly, the app allowed users to return to where they left off after dismissing or addressing the interruption within 15 min of leaving the app; only one CSP participant (1.7%) had to restart data entry after being interrupted:

“Yeah, someone had called me- And I told them I was doing something, I'd call them right back. Interviewer: So you were able to answer the phone and then whenever you- Participant: Mm-hmm (affirmative). And go right back to it” – Participant F, a Group B participant with an Android smartphone talking about interruptions while using BYOD.

#### Were there any data security or safety concerns when using either device type?

Only two CSP participants (3.3%) reported concerns with aspects of data security. One CSP participant (1.7%) was worried that using the PD may allow her location to be “tracked” and another one (1.7%) was worried data from the PRO measures may “cross paths” with other software running on her BYOD. The majority of CSP participants did not raise any concerns regarding data security, while some (PD n = 13, 21.7%; BYOD n = 13, 21.7%) commented there was a general acceptance of some risk when doing any online activity.

#### Which device type did participants prefer to use for completing the PRO measures?

At the second interview (Visit 3), of the 57 LP participants who had used the app on both device types, just under half preferred PD (n = 26, 45.6%) and slightly more than half preferred BYOD (n = 29, 50.9%). Two LP participants (3.5%) had no preference. The most common reason for preferring PD was that it was “dedicated” to the study (n = 17, 29.8%). Another prominent reason for preferring the PD was the lack of interruptions from messages/calls (n = 7, 12.3%). The “convenience” of carrying a single device was the main reason for preferring BYOD (n = 25, 43.9%). Those LP participants who preferred BYOD also reported that it was because they were more familiar with their own device than the PD (n = 13, 22.8%).

“I like the [PD] because I don't have to fool around. I don't have to miss any calls, you know, or anything like that. I can just focus on that device..” – Participant G, a Group B participant with an Android smartphone talking about PD

“I prefer the [PD] because when I look at the [PD], it reminds I have to do it. And the reason is, because the phone I'm not familiar with. ..If I would have looked at the other phone [PD], I would have thought, "Oh, I've gotta do this." I'd feel better, more comfortable doing it with the [PD].” – Participant H, a Group B participant with an Android smartphone talking about PD

“I would rather have my phone.…I mean that way I don't have to keep up with somebody's else's stuff and my phone stays right beside me all the time. …I don't have to keep up with several different devices.” – Participant B, a Group B participant with an iOS smartphone talking about BYOD

“Because it's with me all the time. It's not an extra thing to remember during the course of the day if I were going out one evening - "Oh my god, I forgot my test phone - I don't have to separately charge it.” – Participant C, a Group A participant with an iOS smartphone talking about BYOD

Interestingly, it was found that there was a significant difference in device preference between LP participants in Group A and Group B, with both groups preferring the device they used in the second study period (*p* = 0.0076). There was no statistical difference when device preference was examined according to type of BYOD ownership (iOS versus Android). In addition, all nine participants who experienced technical issues with the PD, and were therefore switched to Group B, completed the second interview (Visit 3) and were included in the LP. When asked for their preference for either device, five of them (8.8%) preferred PD, while three participants (5.3%) preferred BYOD, and one participant (1.8%) stated no preference for either device type. Thus, there was not a greater preference for BYOD due to the technical issues with PD as may have been expected.

## Discussion

These qualitative data demonstrated that participants’ experience of completing the PRO measures was largely positive and consistent across both PD and BYOD, with preference for one of the device types somewhat evenly split. This finding provides support that BYOD is as acceptable to study participants as PD for PRO data collection. Some participants specifically liked the “dedicated” purpose of the PD, whereas others liked the “convenience” of BYOD, which underpinned participants’ device preference.

Previous quantitative research reported that 45% of study participants preferred BYOD [3]. Our results are somewhat more positive toward BYOD with 50.9% of our participants (29/57) preferring BYOD. However, the quantitative research by Byrom et al. (2018) also reported that 40% of their participants indicated they had no preference for either BYOD or PD. In contrast, we found that only two participants had no preference between device types and the remaining participants (45.6%; 26/57) preferred PD [3]. Unlike Byrom et al. (2018) and Pugliese et al. (2016), our study involved a cross-over repeated measures design (as opposed to a single-day assessment at a research site) [3] that allowed participants to consider more comprehensively the relative advantages and disadvantages of both device types in the context of daily data collection. Our study also included a larger qualitative sample to better understand experiences of using both device types. One implication of our study’s findings may be to offer participants enrolling in clinical research the opportunity to choose whether they use their own smartphone or a PD, where appropriate, allowing their device preferences to be met.

The number of missing days reported by participants was similar across device types—a finding supported by our quantitative analyses reported separately. Reasons for missingness appear to be attributed to various factors including lifestyle conflicts, forgetfulness, poor health, and technical issues. This reinforces the importance of proactive monitoring by the site to supplement the alarms and reminders provided by the ePRO technology, and patient-facing help desk support accessible within diary completion windows for ePRO devices, both BYOD and PD, to ensure any technical difficulties do not impact completion rates.

The requirement for technical support from the ePRO provider’s help desk was a key learning from this study. Of note, a few participants reported that the Help Desk in this study was not able to resolve their issue because the completion window timed out, assistance was not given, or they were asked to call back the next day. Such experiences could prevent participants from reaching out for external support in similar circumstances in the future. Again, this emphasizes the need for accessible patient-facing help desk support throughout a clinical study when utilizing PD or BYOD. Furthermore, there were some instances where technical issues with the PD prohibited enrollment to PD, meaning participants had to start the study using their own smartphone. It is of critical importance that the ePRO provider confirms all PDs are operating correctly before distributing them to sites as well as ensuring site staff reconfirm they are fully functional prior to the study enrollment visit.

Although most participants found the reminders useful on both device types, the fact that several participants reported not receiving them (or being unsure if they did) is concerning because reminders were important for maintaining participant compliance. Therefore, it is essential that any alarms used are sufficiently loud and visual to ensure participants are aware of them. When using their own smartphones, most participants did not experience any device-related interruptions. For the small number who did receive a phone call or text message during completion, there was no impact on completing the PRO measures, suggesting such factors may not impact compliance or data accuracy in this population.

One of the concerns surrounding the use of BYOD is that it may eliminate potential participants from a clinical trial due to the technical/device requirements of their own smartphone to meet the criteria for eligibility [1]. We monitored the reasons for non-participation among the 59 individuals who were approached for participation but were unable to participate. Of these 59, just 14 could not do so because of BYOD-related reasons—either not owning a smartphone (n = 9) or not wanting to use their smartphone in this study (n = 5). This number represents a small percentage of the total participants approached to be in this study (i.e., 14 out of 123, or 11.4%), suggesting that device-related reasons are unlikely to be a major concern for trial recruitment. Nonetheless, it does reflect the reality that a certain percentage of potential study participants may be unable or unwilling to use their own smartphone, and that a certain degree of provisioning will be necessary to ensure that trial recruitment is not biased. There were some limitations to this study. This study was relatively short (i.e., roughly four weeks); during a longer study, participants may encounter other issues related to their own smartphone such as software updates or replacing their smartphone. ePRO vendors would need to ensure their apps are compatible with future operating system updates and research protocols would need to include mechanisms for participants to report when they are temporarily without a smartphone and for potential alternative provisions to be made (e.g., supply of PD, other internet-enabled data capture sources). In addition, the generalizability of the results may be limited due to the age of the study sample, which started at age 40 as part of the COPD diagnostic criteria, as well as all participants being from the US. The results may have been different if younger participants, as well as those from other countries or cultures, had been included in the study.

## Conclusion

Overall, our study demonstrates the feasibility of BYOD among older participants with a chronic health condition, at least for a short period of time; this finding is encouraging in regard to the potential for wider use of BYOD across a range of ages and with other PRO measures. This study supports the use of BYOD as a potential addition to PD for collecting PRO data in COPD studies and contributes evidence that BYOD may be employed to collect PRO data in demographically diverse patient populations. Finally, the findings from this study encourage continued testing and utilization of new approaches for PRO data that help reduce operational costs, provide greater efficiency within clinical trials, and remove unnecessary burden from respondents.

## Data Availability

Data is available from corresponding author upon reasonable request.
